# A narrative review of the role of renal artery intervention in renovascular hypertension

**DOI:** 10.3389/fsurg.2025.1712028

**Published:** 2025-12-15

**Authors:** Li Che, Zilong Wang

**Affiliations:** 1 Department of Cardiology, Central Hospital of Dalian University of Technology, Dalian, China; 2 Department of Neurosurgery, The First People’s Hospital of JinZhou District, Dalian, China

**Keywords:** angioplasty, hypertension, renal artery obstruction, renovascular, treatment outcome

## Abstract

Renovascular hypertension is a form of secondary hypertension caused by renal artery stenosis and often shows a limited response to medical treatment. Over recent years, renal artery interventions, primarily angioplasty and stenting, have been increasingly used as treatment options in selected patients. This narrative review summarizes current techniques, clinical outcomes, and evidence related to renal artery interventions in the management of renovascular disease. It also highlights existing knowledge, challenges, emerging technologies, and future directions for improving patient selection, procedural safety, and long-term effectiveness of intervention strategies. By consolidating recent developments and identifying critical knowledge gaps, this review provides an updated and practical overview for clinicians and offers guidance for future research in the field of renal artery intervention.

## 1 Introduction

Renovascular hypertension (RVH) refers to hypertension caused by renal artery disease. It occurs in 1%–5% of individuals with hypertension and is the leading cause of secondary hypertension (1). Atherosclerosis and fibromuscular dysplasia (FMD) are the two most common etiologies of RVH. These conditions cause renal artery stenosis (RAS), which results in renal ischemia and activation of the renin–angiotensin–aldosterone system, thereby elevating blood pressure (BP) (2). Because medical treatment is not always effective in these patients, alternative therapeutic options may be required (3). Percutaneous transluminal angioplasty and stent implantation are two such options (4).

The pathophysiology of RVH is complex. RAS results in reduced renal perfusion, leading to increased renin release from juxtaglomerular cells, elevated angiotensin II levels, and subsequent increases in BP (5). Renal ischemia can also lead to deteriorating renal function and chronic kidney disease (6). Therefore, it is important to identify hypertension at an early stage to prevent further renal damage and cardiovascular complications (7).

Recent studies have shown the potential benefits of renal artery interventions in patients with RVH. For instance, percutaneous transluminal angioplasty can improve renal blood flow and reduce BP in patients who have severe stenosis and relatively preserved renal function (8). Stenting may also help control BP in patients with long-term restenosis (9). However, the results of these interventions may vary depending on the etiologies of RAS, existing diseases, and the degree of renal dysfunction (10–12). Despite their potential benefits, renal artery interventions also carry risks. Complications may include renal artery dissection, thrombosis, and contrast-induced nephropathy (6, 13, 14). Therefore, careful patient selection and thorough preoperative assessment are essential. Additional research is also needed to establish clear guidelines for the use of these interventions in different patient populations.

In summary, renal artery intervention is an effective treatment option when medical therapy fails to control BP in RVH. Correcting the underlying vascular pathology can help control BP and preserve renal function. As our understanding of the pathophysiology of RVH advances, our therapeutic approaches will continue to improve. Future studies should focus on patient selection, procedural techniques, and long-term outcomes.

## 2 Basic concepts of renal artery interventional therapy

### 2.1 Definition and classification of RAS

RAS is defined as the narrowing of one or both renal arteries. It may lead to secondary hypertension and renal insufficiency. Atherosclerosis is more common in older individuals, whereas FMD typically affects younger women. RAS may be classified according to its cause, the location of the lesion, and the degree of stenosis (1). This classification is important for determining appropriate treatment strategies in clinical practice.

Research shows that atherosclerotic RAS accounts for more than 90% of all cases, and these patients often have hypertension, diabetes, and other cardiovascular risk factors (15, 16). FMD is a nonatherosclerotic and noninflammatory arterial disease that primarily affects the renal and carotid arteries. It is more prevalent in young women and may result in hypertension (17). Therefore, understanding the classification of RAS is essential for determining the most suitable therapeutic approach, as the cause and severity of stenosis influence treatment decisions and outcomes.

### 2.2 Basic principles of interventional surgery

Interventional therapies for RAS aim to restore blood flow to the kidneys and lower BP. Common techniques include angioplasty and stenting (18). Angioplasty involves inserting a catheter with a balloon at its tip into the vessel and inflating the balloon at the stenotic area to widen the artery (19). If there is a high risk of re-stenosis, a stent may be implanted to maintain arterial patency. Interventional therapy requires careful RAS patient selection, image guidance (usually fluoroscopy), and sterile technique to minimize risks such as infection or thrombosis (20). Another important consideration is the clinical time of intervention, which is often performed for patients with resistant hypertension or worsening renal function (21).

### 2.3 Indications and contraindications for surgery

Indications for renal artery revascularization primarily include RVH, particularly when refractory to medical therapy, and significant chronic renal failure due to stenosis. Patients with atherosclerosis, especially those with unilateral RAS and preserved renal function, are frequently considered for revascularization.

Contraindications include comorbidities such as severe heart failure or significant renal insufficiency, which may limit the anticipated benefit of intervention (22, 23). Patients with extensive atherosclerotic disease involving multiple vascular beds are often unlikely to benefit from renal artery intervention because the probability of improving BP or renal function is low (1). As stent and balloon technologies continue to evolve, more favorable outcomes may be expected in the future (24).

In summary, RAS is a significant clinical condition that requires appropriate recognition and management. The main concepts of interventional therapy, including definition, classification, and surgical principles, provide the foundation for managing these patients. Understanding the indications and contraindications helps identify the appropriate patient population for intervention and supports optimal treatment outcomes.

## 3 Clinical effectiveness of renal artery angioplasty

### 3.1 Surgical methods and implementation steps

Renal artery angioplasty is a minimally invasive procedure used to treat RAS (25). The procedure typically involves catheterization through the femoral artery, followed by advancement of the catheter into the renal artery under imaging guidance. A balloon at the catheter tip is then inflated at the stenotic segment to widen the artery. This restores adequate renal circulation, which helps control BP and prevents further kidney damage.

The procedure is performed under local anesthesia and sedation. Most patients recover quickly and experience little postoperative pain. Key steps include using preoperative imaging, renal artery catheterization, balloon inflation, and post-procedure imaging. The use of intravascular ultrasound (IVUS) has improved the accuracy of lesion detection, enhancing procedural outcomes (8).

### 3.2 Postoperative blood pressure changes assessment

Monitoring BP changes is important to evaluate how well the procedure worked after renal artery angioplasty. Many patients experience measurable reductions in BP. One study reported an average decrease of 14/9 mmHg at one month, with the effect sustained for up to one year (26). Patients often require fewer antihypertensive medications following the surgery (27). Assessment involves regular BP monitoring and evaluation of renal function after treatment. While BP may normalize in some individuals, others may simply require fewer medications. The overall goal is to restore normal renal perfusion and reduce long-term cardiovascular risks.

### 3.3 Complications and management

Renal artery angioplasty is generally safe, but complications can occur. Adverse events may lead to acute kidney injury or necessitate additional interventions, including surgical repair or repeat angioplasty. Management involves close monitoring of renal function and BP, as well as imaging to detect complications. Renal artery occlusion may require urgent treatment, including renal autotransplantation in high-risk cases (6). Post-angioplasty antiplatelet therapy is mandatory to reduce the risk of thrombosis.

Patient education regarding warning signs and symptoms of complications and adherence to follow-up appointments is crucial. While the risk of complications exists, angioplasty offers significant benefits in appropriately selected patients with RVH, and these typically outweigh the associated risks (28).

## 4 Application of stent implantation in hypertension treatment

### 4.1 Types of stents and their indications

Stent implantation is widely used to treat RAS-induced hypertension. RAS resulting from atherosclerosis or FMD may lead to secondary hypertension. Two major stent types are available: Bare-metal stents (BMS) and drug-eluting stents (DES). BMS are technically easier to deploy and more cost-effective, whereas DES are designed to reduce restenosis by inhibiting smooth muscle cell proliferation through drug elution (29).

The choice of stent depends on the underlying cause of RAS, renal artery anatomy, and comorbidities. In nonatherosclerotic disease such as FMD, balloon angioplasty alone is often effective, though stent placement may be required in selected cases (30, 31). In atherosclerotic RAS, the more complex vascular anatomy and higher complication rates often necessitate stenting as a preferred option (32).

Stent placement aims not only to control hypertension but also to prevent renal ischemia and preserve renal function. Even in patients with resistant hypertension, successful stenting may result in BP reduction, particularly in those with severe but asymptomatic stenosis (33). Hence, the type of stent selected and the decision to stent are important contributors to optimal hypertension management.

### 4.2 Postoperative outcomes and long-term follow-up

Stent implantation generally yields favorable outcomes; many studies show significant reductions in BP and improvements in renal function. Initial technical success rates often exceed 90%, and most patients report relief of hypertensive symptoms (16). Long-term follow-up is necessary to evaluate the intervention's durability and to detect the occurrence of late complications such as restenosis.

In one cohort, long-term BP control was maintained in some patients, although a subset developed recurrent hypertension (34). The rate of in-stent restenosis varies by stent type. For example, among 398 patients with chronic heart disease who underwent percutaneous coronary intervention with sirolimus-eluting stents (SES), the overall one-year restenosis rate was 9.3% (35). Factors such as age, hypertension, diabetes, increased low-density lipoprotein cholesterol, and lesions targeting left circumflex arteries affect the restenosis risk associated with the use of SES. Regular imaging and clinical follow-up can facilitate early detection of such a possible event (35).

Studies also show that long-term survival among patients undergoing renal artery stenting is comparable to that of medically treated individuals, especially in high-risk groups (36). This suggests that stenting may improve both BP control and overall cardiovascular health. However, patient selection is important as patients with high comorbidities or advanced renal disease may not benefit from the intervention (8).

In conclusion, stent implantation is a valuable treatment option for hypertension related to RAS. Its long-term effectiveness depends on careful patient selection and continuous clinical follow-up.

### 4.3 Analysis of stent-related complications

Although stenting is effective, it can be associated with complications. These can be classified as immediate or delayed and often relate to technical aspects of the procedure. Immediate complications include access-site bleeding, vascular complications, and acute renal failure, particularly in patients with pre-existing renal insufficiency (23, 37, 38). Minimizing these risks requires careful procedural planning and skilled operators.

Chronic complications have important implications for long-term outcomes. Restenosis is the most common delayed complication, usually caused by neointimal hyperplasia or, less frequently, late stent thrombosis (39). Factors affecting restenosis include stent type, patient vascular biology, and characteristics of the renal artery disease (11, 40, 41).

Other complications include stent migration, fracture, and in-stent thrombosis development (13, 42, 43). These complications may require further interventions such as repeat angioplasty or surgical revision, which increase both clinical complexity and healthcare costs. Although stent implantation is an effective treatment, vigilance for complications remains essential. Continued research on device development and refinement of patient selection criteria is necessary to maximize safety and clinical benefit.

## 5 Latest research findings and progress

Recent renal interventions have been affected by numerous clinical trials. These data show that percutaneous renal artery revascularization is performed mainly for hypertension and impaired renal function (44). One study reported that acute anatomic renal injury may occur after renal artery interventions and is associated with negative long-term outcomes, including reduced survival and increased renal-related morbidity (23). These findings emphasize the need for careful preprocedural planning and patient selection. Guideline-directed medical therapy is essential, especially for patients with refractory symptoms (45). Evidence suggests that patients with significant stenosis are more likely to benefit from stenting. Overall, clinical trials on renal interventions have expanded our understanding of procedural risks and support the refinement of treatment protocols.

### 5.1 Application of new technologies in interventional therapy

New technologies are being incorporated into the interventional therapy of renal artery disease. Three-dimensional (3D) printing technology is among the most promising innovations for renal artery interventions. One study demonstrated that 3D-printed renal artery models help clinicians better understand complex anatomy (46). This technology may minimize the need to use contrast agents and fluoroscopy during procedures. This also improves the efficiency and safety of endovascular treatment. IVUS has been recognized as a valuable tool for optimizing stent placement and assessing vascular lesions (47). Incorporating these imaging techniques represents a significant advancement in the precision of renal interventions.

### 5.2 Exploration of multidisciplinary collaborative models

Multidisciplinary teamwork has become increasingly preferred in the management of complex disease conditions. Evidence indicates that effective communication and collaboration lead to more appropriate treatment strategies and higher levels of patient satisfaction (48). Pharmacists make important contributions within these teams by reducing adverse drug events in older adults with multiple comorbidities and improving overall medication management (49).

In summary, clinical research, advanced technologies, and multidisciplinary collaboration are essential components of modern renal interventions. These factors will continue to influence the development of new care strategies and improvements in patient outcomes.

## 6 Future directions of renal artery interventional therapy

### 6.1 Technological innovations and development trends

The field of renal artery intervention is evolving rapidly. Advances are occurring across multiple domains, including imaging, minimally invasive approaches, and biomaterials. Improvements in imaging techniques provide clearer vascular visualization, enabling more precise treatment planning, particularly for RAS caused by FMD (50).

Treatment strategies are also shifting. For instance, robot-assisted interventions have been shown to improve surgical precision and control during procedures, potentially reducing complications and facilitating faster patient recovery (51). Robotic systems also make the surgeon's job physically easier, helping them stay sharp and avoid fatigue during those long procedures (52). Furthermore, emerging technologies such as bioresorbable stents and drug-coated balloons are gaining attention for their ability to minimize the risk of restenosis by delivering localized medication effectively (53).

Artificial intelligence (AI) and machine learning are proving indispensable in analyzing patient data and medical imaging. These tools predict patient responses to treatments by identifying patterns in patient data and medical imaging from historical information, enabling physicians to develop more personalized care strategies. Such AI-assisted analysis has been recently shown to enhance patient outcomes while optimizing resource allocation (54).

Overall, renal artery intervention is undergoing a significant transformation. Emerging technologies are enabling more accurate diagnostics and safer procedural techniques, leading to better patient outcomes. As these innovations continue to advance and integrate, treatments are expected to become more effective and less invasive.

### 6.2 Research prospects for personalized treatment

There is a growing shift toward personalized medicine in renal artery interventions. Recent findings suggest that tailoring treatment strategies to individual patient characteristics, such as age, sex, and comorbidities, can significantly enhance treatment efficacy (55). This emphasizes the importance of developing patient-specific treatment plans (1).

Research on genetic and molecular markers related to renal artery disease is expanding. Understanding genetic factors may lead to more targeted therapeutic options. For instance, identifying mutations in genes regulating vascular smooth muscle cell activity could assist in identifying patients at high risk for severe disease. This would allow clinicians to deliver more personalized treatments.

Pharmacogenomics also offers opportunities for personalized treatment. Clinicians may eventually tailor antihypertensive therapy based on genetic variations that influence drug metabolism and response. This is particularly relevant for RVH, as responses to standard antihypertensive medications vary among patients.

The development of personalized therapy in renal artery intervention also depends on advanced intraprocedural imaging. Techniques such as IVUS and optical coherence tomography provide real-time, high-resolution visualization of vessel anatomy and functional status. These tools allow clinicians to adapt and optimize the treatment plan based on an individual's immediate response during the procedure itself (56).

Future research in personalized renal artery intervention is promising. Investigations focused on genetic profiling, response prediction, pharmacogenomics, patient-oriented models of care, and advanced imaging are anticipated. Personalized approaches are expected to increase treatment precision, reduce complications, and improve outcomes for patients with renal artery disease.

## 7 Conclusion

Interventional therapy to treat RAS has become an important option for patients with secondary hypertension who do not respond to medical therapy. This article reviewed recent advancements in this field, including both accomplishments and remaining challenges.

The management of RVH has transformed significantly due to the advancements in interventional techniques. The progression from percutaneous transluminal renal angioplasty to stenting has been widely studied. Many studies report that these interventions can effectively reduce BP and improve renal function in selected patients. However, several randomized trials report conflicting results. For example, one study found that the BP improvement after stent implantation diminished after six months (57). In the ASTRAL randomized trial, revascularization plus medical therapy showed no significant benefit in systolic BP compared with medical therapy alone, while 23 patients experienced serious procedural complications, including two deaths and three amputations (58). In the STAR trial, 10 of 64 patients in the stenting plus medical treatment group (46 completed stenting) and 16 of 76 patients in the medical treatment alone group reached the primary end point of a decrease of ≥20% in creatinine clearance. However, no significant differences were seen between the two groups in the primary or most secondary endpoints, despite the occurrence of complications in the stent group (59). Similarly, the CORAL trial reported no statistically significant differences in primary endpoint outcomes between the stent plus medical therapy group and the medical therapy-only group (60). These findings demonstrate the need for careful patient selection and adherence to rigorous follow-up protocols.

Given the diversity and complexity of patient presentations, defining optimal treatment strategies can be challenging. Clinicians and researchers must proceed cautiously, with a clear understanding of RVH pathophysiology and strong evidence from well-designed clinical trials. Continued collaborative efforts are needed to establish best practices and consensus guidelines.

The future of renal artery intervention lies in personalized care. Tailoring treatment to individual characteristics, comorbidities, and preferences will be essential. Integration of advanced imaging technologies and biomarkers will be critical for identifying the most suitable treatment options. Emphasis on long-term follow-up is necessary to assess the durability and safety of interventions.

Innovations such as drug-coated stents and bioresorbable scaffolds hold significant promise for improving long-term results. Integration of these advancements with treatment strategies may reduce restenosis and improve safety. Although emerging approaches such as bioresorbable stents show promise in reducing restenosis rates, their long-term effectiveness remains under investigation.

Renal artery angioplasty and stenting remain promising therapeutic options, but they are underused in clinical practice. A coordinated movement toward personalized medicine will be essential to address the complex interactions between disease mechanisms and treatment responses. Ensuring that each patient receives the safest and most effective therapy remains the guiding goal, and progress in technology and research continues to bring this goal closer.

## References

[1] Dong M Pan D Li J An Y Dong R Zhang J Renal artery reconstruction for refractory hypertension caused by congenital renal artery deficiency. Ann Vasc Surg. (2021) 77:352 e1–e5. 10.1016/j.avsg.2021.05.062 34461240

[2] Persu A Canning C Prejbisz A Dobrowolski P Amar L Chrysochou C Beyond atherosclerosis and fibromuscular dysplasia: rare causes of renovascular hypertension. Hypertension. (2021) 78(4):898–911. 10.1161/HYPERTENSIONAHA.121.17004 34455817 PMC8415524

[3] de Oliveira Campos JL Bitencourt L Pedrosa AL Silva DF Lin FJJ de Oliveira Dias LT Renovascular hypertension in pediatric patients: update on diagnosis and management. Pediatr Nephrol. (2021) 36(12):3853–68. 10.1007/s00467-021-05063-2 33851262

[4] Gunawardena T Sharma H. Transplant renal artery stenosis: current concepts. Exp Clin Transplant. (2022) 20(12):1049–57. 10.6002/ect.2022.0334 36718003

[5] Januszewicz A Januszewicz M Prejbisz A Dobrowolski P Persu A Kreutz R. Atherosclerotic renovascular disease and cardiovascular risk: new concepts. Pol Arch Intern Med. (2022) 132(12):16384–1638. 10.20452/pamw.16384 36533812

[6] Wyatt N Melhem N Booth C Newton J Karunanithy N Sallam M Successful emergency renal auto-transplantation in a child with renovascular disease. J Hypertens. (2025) 43(1):168–72. 10.1097/HJH.0000000000003879 39351854

[7] Martelli E Enea I Zamboni M Federici M Bracale UM Sangiorgi G Focus on the most common paucisymptomatic vasculopathic population, from diagnosis to secondary prevention of complications. Diagnostics (Basel). (2023) 13(14):2356. 10.3390/diagnostics13142356 37510100 PMC10377859

[8] Homorodean C Ober MC Spinu M Olinic M Tataru DA Onea HL Outcomes after stenting of renal artery stenosis in patients with high-risk clinical features. Egypt Heart J. (2024) 76(1):4. 10.1186/s43044-024-00435-z 38236490 PMC10796309

[9] Al Mubarak HA Alshalan SM Alkhunaizi NB Alorabi AH Abdulfatah AM. A case report on fibromuscular dysplasia with extrarenal involvement. Cureus. (2023) 15(12):e50358. 10.7759/cureus.50358 38213346 PMC10781897

[10] Rajgopal R Khan A Syed AA. Renal artery stenosis presenting as hypertension with hypokalemia. CMAJ. (2022) 194(36):E1248–E9. 10.1503/cmaj.220091 36122918 PMC9484623

[11] Peng M Ji W Jiang X Dong H Zou Y Song L Selective stent placement versus balloon angioplasty for renovascular hypertension caused by Takayasu arteritis: two-year results. Int J Cardiol. (2016) 205:117–23. 10.1016/j.ijcard.2015.12.006 26730842

[12] Iwasaki T Mishima E Suzuki T Kikuchi K Toyohara T Seiji K Kidney enlargement effect of angioplasty for nonatherosclerotic renovascular disease: reversibility of ischemic kidney. Hypertens Res. (2020) 43(11):1214–21. 10.1038/s41440-020-0473-6 32444857

[13] Marini M Fernandez-Rivera C Cao I Gulias D Alonso A Lopez-Muniz A Treatment of transplant renal artery stenosis by percutaneous transluminal angioplasty and/or stenting: study in 63 patients in a single institution. Transplant Proc. (2011) 43(6):2205–7. 10.1016/j.transproceed.2011.06.049 21839234

[14] Zhao JH Cheng QL Zhang XY. Long-term follow-up of elderly patients with atherosclerotic renal artery stenosis treated by percutaneous transluminal angioplasty with stent implantation. Zhonghua Yi Xue Za Zhi. (2011) 91(24):1673–6. 10.3760/cma.j.issn.0376-2491.2011.24.005 21914314

[15] Gunawardena T. Atherosclerotic renal artery stenosis: a review. Aorta (Stamford). (2021) 9(3):95–9. 10.1055/s-0041-1730004 34638148 PMC8598311

[16] Drachman DE Metzger DC Jain A Sachar R El-Sayed Abbas A Rosenfield K *De Novo* atherosclerotic renal artery stenosis covered stent treatment for resistant hypertension (ARTISAN) results. J Soc Cardiovasc Angiogr Interv. (2024) 3(12):102400. 10.1016/j.jscai.2024.102400 39807237 PMC11725122

[17] Heredia-Torres N Nieto-Vega FA Rodriguez Azor B Martinez-Rivera V. Fibromuscular dysplasia: an uncommon cause of arterial hypertension. An Pediatr (Engl Ed). (2024) 100(4):e17–e8. 10.1016/j.anpede.2024.03.027 38580586

[18] Kim S Kim MJ Jeon J Jang HR Park KB Huh W Effects of percutaneous angioplasty on kidney function and blood pressure in patients with atherosclerotic renal artery stenosis. Kidney Res Clin Pract. (2019) 38(3):336–46. 10.23876/j.krcp.18.0148 31234613 PMC6727892

[19] Derakhshesh MI Joye E Yager N. Unilateral renal artery stenosis causing hypertensive flash pulmonary oedema. BMJ Case Rep. (2021) 14(9):e244402. 10.1136/bcr-2021-244402 34511412 PMC8438749

[20] Furusawa EA Esposito CSL Honjo RS Suzuki L Leal GN Kim CA Diagnosis and management of systemic hypertension due to renovascular and aortic stenosis in patients with Williams-Beuren syndrome. Rev Assoc Med Bras. (2018) 64(8):723–8. 10.1590/1806-9282.64.08.723 30673043

[21] Wang X Zhou Z Li J Su G Li X. Hypertension as a prominent manifestation secondary to renal artery lesions in pediatric Behcet's disease. Pediatr Rheumatol Online J. (2024) 22(1):19. 10.1186/s12969-023-00932-6 38243321 PMC10797725

[22] Fezzi S Castaldi G Widmann M Ruzzarin A Tavella D Ribichini F. Integrated anatomical and functional approach for tailored renal interventions-in patients with resistant arterial hypertension. J Nephrol. (2022) 35(6):1747–52. 10.1007/s40620-022-01261-9 35157245

[23] Cheun TJ Davies MG. The implications of acute anatomic injury after percutaneous renal intervention. J Endovasc Ther. (2024):15266028241268826. 10.1177/15266028241268826 39129419

[24] Yesiltas MA Koyuncu AO Ak HY Haberal I. Endovascular treatments of atherosclerotic renovascular disease: a narrative review and literature search. J Int Med Res. (2023) 51(10):3000605231206057. 10.1177/03000605231206057 37882729 PMC10605686

[25] Zhao L Zhao X Hu X Yang H Wu L. Midterm outcomes of angioplasty for pediatric renovascular hypertension. J Vasc Interv Radiol. (2022) 33(4):399–407. 10.1016/j.jvir.2021.10.035 34896573

[26] Courand PY Dinic M Lorthioir A Bobrie G Grataloup C Denarie N Resistant hypertension and atherosclerotic renal artery stenosis: effects of angioplasty on ambulatory blood pressure. A retrospective uncontrolled single-center study. Hypertension. (2019) 74(6):1516–23. 10.1161/HYPERTENSIONAHA.119.13393 31656101

[27] Li P Yang X Niu G Yan Z Zhang B Yang M. Percutaneous transluminal renal angioplasty for pediatric hypertension secondary to total renal artery occlusion. J Vasc Interv Radiol. (2024) 35(9):1332–9. 10.1016/j.jvir.2024.03.011 38499268

[28] Goodman J Kulkarni S Selvarajah V Hilliard N Russell N Wilkinson IB. Renal autotransplantation for uncontrolled hypertension in nonatherosclerotic renal artery stenosis-2 case reports and a brief review of the literature. Hypertension. (2024) 81(4):669–75. 10.1161/HYPERTENSIONAHA.123.19878 38507507

[29] Soetomo CT Wardhana DPW Gunawan MFB Satyarsa ABS Rosyidi RM. Elucidating the benefit of drug-eluting stent for symptomatic intracranial atherosclerotic stenosis: meta-analysis of randomized controlled trials. Surg Neurol Int. (2025) 16:363. 10.25259/SNI_775_2025 41036046 PMC12482777

[30] Edem E Aksoy MN Pabuccu MT Tatli E. Endovascular treatment of renal artery stenosis due to fibromuscular dysplasia—is stent implantation underused in this circumstance? Heart Views. (2016) 17(2):69–71. 10.4103/1995-705X.185118 27512536 PMC4966212

[31] Skeik N Hyde JR Olson SL Thaler CM Abuatiyeh W Ahmed AK Nonatherosclerotic abdominal vasculopathies. Ann Vasc Surg. (2019) 60:128–46. 10.1016/j.avsg.2019.04.004 31200053

[32] Louis DW Kolte D Kennedy K Lima FV Abbott JD Shemin D Thirty-day readmission after medical versus endovascular therapy for atherosclerotic renal artery stenosis. Am J Cardiol. (2020) 125(7):1115–22. 10.1016/j.amjcard.2019.12.042 32005439

[33] Qiu C Shao J Liu X Liu B. Utilizing flat-panel detector parenchymal blood volume imaging (FD-PBV) for quantitative kidney perfusion analysis during the process of percutaneous transluminal renal angioplasty (PTRA): a case report. Medicine (Baltimore). (2017) 96(47):e8654. 10.1097/MD.0000000000008654 29381939 PMC5708938

[34] Davies MG Saad WE Peden EK Mohiuddin IT Naoum JJ Lumsden AB. The long-term outcomes of percutaneous therapy for renal artery fibromuscular dysplasia. J Vasc Surg. (2008) 48(4):865–71. 10.1016/j.jvs.2008.05.030 18692345

[35] Zhao J Wang X Wang H Zhao Y Fu X. Occurrence and predictive factors of restenosis in coronary heart disease patients underwent sirolimus-eluting stent implantation. Ir J Med Sci. (2020) 189(3):907–15. 10.1007/s11845-020-02176-9 31989420

[36] Modrall JG Zhu H Prasad T Moe O Dworkin LD Cutlip DE Retrieval of renal function after renal artery stenting improves event-free survival in a subgroup analysis of the cardiovascular outcomes in renal atherosclerotic lesions trial. J Vasc Surg. (2023) 77(6):1685–92 e2. 10.1016/j.jvs.2022.12.067 36736864

[37] Tigen MK Ozdil MH Cincin A Gurel E Sunbul M Sahin A Bleeding risk with concomitant use of tirofiban and third-generation P2Y12 receptor antagonists in patients with acute myocardial infarction: a real-life data. Anatol J Cardiol. (2021) 25(10):699–705. 10.5152/AnatolJCardiol.2021.27974 34622784 PMC8504671

[38] Zhang W Schneider M Zartner P. Follow-up of hypertension after aortic coarctation stent implantation based on safe and effective re-intervention: a retrospective cohort study. J Thorac Dis. (2022) 14(10):3924–33. 10.21037/jtd-22-1134 36389339 PMC9641353

[39] Lindholm D James S. Bioresorbable stents in PCI. Curr Cardiol Rep. (2016) 18(8):74. 10.1007/s11886-016-0750-9 27312934

[40] Zhang J Zhang Q Zhao K Bian YJ Liu Y Xue YT. Risk factors for in-stent restenosis after coronary stent implantation in patients with coronary artery disease: a retrospective observational study. Medicine (Baltimore). (2022) 101(47):e31707. 10.1097/MD.0000000000031707 36451388 PMC9704915

[41] Baktashian M Saffar Soflaei S Kosari N Salehi M Khosravi A Ahmadinejad M Association of high level of hs-CRP with in-stent restenosis: a case-control study. Cardiovasc Revasc Med. (2019) 20(7):583–7. 10.1016/j.carrev.2018.08.015 30232022

[42] Badero OJ Adetiloye AO Osibowale B. Common iliac artery stent migration post intervention: a case report and percutaneous management options. JACC Case Rep. (2025) 30(24):104680. 10.1016/j.jaccas.2025.104680 40846384 PMC12371380

[43] Zhou TY Wang HL Tao GF Chen SQ. Stent fracture after transjugular intrahepatic portosystemic shunt: a case report. World J Gastrointest Surg. (2025) 17(5):104893. 10.4240/wjgs.v17.i5.104893 40502521 PMC12149923

[44] Arab SF Alhumaid AA Abu Alnasr MT Altuwaijri TA Al-Ghofili H Al-Salman MM Review of renal artery stenosis and hypertension: diagnosis, management, and recent randomized control trials. Saudi J Kidney Dis Transpl. (2022) 33(1):147–59. 10.4103/1319-2442.367807 36647988

[45] Bob-Manuel T White CJ. Renal and mesenteric artery intervention. Interv Cardiol Clin. (2020) 9(2):169–85. 10.1016/j.iccl.2019.11.002 32147118

[46] Memon S Friend E Kalra S Janzer S George JC. 3D printing of renal arteries for endovascular interventions: feasibility, utility, and correlation with renal arteriograms. J Invasive Cardiol. (2021) 33(12):E986–E92. 10.25270/jic/21.00041 34866051

[47] Bob-Manuel T Amoran OE Jenkins C Obafemi O Tutor A Tafur J. Renal interventions in the management of hypertension. Curr Opin Cardiol. (2021) 36(4):444–52. 10.1097/HCO.0000000000000859 33929362

[48] Barry ES Varpio L Teunissen P Vietor R Kiger M. Preparing military interprofessional health care teams for effective collaboration. Mil Med. (2025) 190(3-4):e804–e10. 10.1093/milmed/usae515 39508535

[49] Gemmeke M Taxis K Bouvy ML Koster ES. Perspectives of primary care providers on multidisciplinary collaboration to prevent medication-related falls. Explor Res Clin Soc Pharm. (2022) 6:100149. 10.1016/j.rcsop.2022.100149 35755717 PMC9218163

[50] Khan A Raskin D Partovi S Kirksey L. Role of multimodality imaging pre-access for planning of surgical creation of arteriovenous fistulas and arteriovenous grafts in the chronic kidney disease and end-stage renal disease population. Int J Cardiovasc Imaging. (2025). 10.1007/s10554-025-03356-3 PMC12847075 40272683

[51] Gil RJ Beyar R Haude M. A brief history and clinical use of robotic procedures in the cardiovascular system. Kardiol Pol. (2025) 83(3):262–8. 10.33963/v.phj.105099 40078083

[52] Cha JS Fuller P Ball M Samreen S Sylla P Stefanidis D SAGES tool for assessing robotic surgery systems (STARSS). Surg Endosc. (2025) 39(9):5479–91. 10.1007/s00464-025-11897-w 40676302 PMC12408702

[53] Tannu M Jones WS Swaminathan RV Rymer JA Gutierrez JA. Femoropopliteal endovascular intervention: a review of the current landscape. Circ Cardiovasc Interv. (2025) 18(5):e014024. 10.1161/CIRCINTERVENTIONS.124.014024 40276857

[54] Weerarathna IN Kamble AR Luharia A. Artificial intelligence applications for biomedical cancer research: a review. Cureus. (2023) 15(11):e48307. 10.7759/cureus.48307 38058345 PMC10697339

[55] Ottoni M Nicoletta F Pederzani A Barone A Ridolo E. Personalization of therapy for patients with eosinophilic esophagitis. Expert Rev Clin Immunol. (2025) 21(10):1373–82. 10.1080/1744666X.2025.2565663 40988499

[56] Kang Y Wu Q Xu J Hong M Ma Y Tang X Intravascular ultrasound provides additional insights in the hypertensive patients with focal renal artery fibromuscular dysplasia. Hypertens Res. (2023) 46(6):1407–16. 10.1038/s41440-023-01216-y 36765108

[57] Arthurs Z Starnes B Cuadrado D Sohn V Cushner H Andersen C. Renal artery stenting slows the rate of renal function decline. J Vasc Surg. (2007) 45(4):726–32. 10.1016/j.jvs.2006.12.026 17398382

[58] Investigators A Wheatley K Ives N Gray R Kalra PA Moss JG Revascularization versus medical therapy for renal-artery stenosis. N Engl J Med. (2009) 361(20):1953–62. 10.1056/NEJMoa0905368 19907042

[59] Bax L Woittiez AJ Kouwenberg HJ Mali WP Buskens E Beek FJ Stent placement in patients with atherosclerotic renal artery stenosis and impaired renal function: a randomized trial. Ann Intern Med. (2009) 150(12):840–8. 10.7326/0003-4819-150-12-200906160-00119 19414832

[60] Murphy TP Cooper CJ Matsumoto AH Cutlip DE Pencina KM Jamerson K Renal artery stent outcomes: effect of baseline blood pressure, stenosis severity, and translesion pressure gradient. J Am Coll Cardiol. (2015) 66(22):2487–94. 10.1016/j.jacc.2015.09.073 26653621 PMC4819253

